# Overcoming Fear of Flying: A Combined Approach of Psychopharmacology and Gradual Exposure Therapy

**DOI:** 10.7759/cureus.39773

**Published:** 2023-05-31

**Authors:** Athena Beth V Abuso, Muneeb Hashmi, Hamdaan Hashmi, Anselm Khoo, Ajay Parsaik

**Affiliations:** 1 Faculty of Medicine and Surgery, University of Santo Tomas, Manila, PHL; 2 Department of Computer Science & Engineering, Texas A&M University, College Station, USA; 3 Department of Entomology, Texas A&M University, College Station, USA; 4 Department of Clinical Services, Westpark Springs Behavioral Hospital, Richmond, USA; 5 Department of Psychiatry, Marshfield Clinic Health System, Marshfield, USA

**Keywords:** gradual exposure therapy, selective serotonin reuptake inhibitors, beta blockers, benzodiazepines, fear of flying, aviophobia

## Abstract

Aviophobia, the fear of flying, is a prevalent type of situational-specific phobia categorized under anxiety disorders in The Diagnostic and Statistical Manual of Mental Disorders, Fifth Edition (DSM-5). Patients with aviophobia experience intense, irrational fear when traveling by air. Active avoidance of the phobic stimulus is a diagnostic feature that affects one's quality of life and commonly leads to significant functional limitations. Virtual reality based gradual exposure therapy is a treatment option for aviophobia due to its easy accessibility and low cost, but it may not be very effective. This case reports the effectiveness of using psychopharmacologic treatment in combination with real-life gradual exposure therapy to successfully treat a patient with aviophobia. Written consent from the patient was obtained prior to the writing and submission of this case report.

## Introduction

Aviophobia, or the fear of flying, is a prevalent type of anxiety disorder that can significantly impair a patient’s personal and social life. Consequences from aviophobia can range from feelings of embarrassment to relationship and career limitations [1]. The Diagnostic and Statistical Manual of Mental Disorders, Fifth Edition (DSM-5) classifies aviophobia as a situational-specific phobia. The diagnostic criteria for specific phobias indicate that people suffering from aviophobia experience marked fear or anxiety about flying, and the fear or anxiety experienced is out of proportion to the actual danger, lasting for six or more months [2]. It is estimated that approximately 2.5%-40% of the population of Western and Northern Europe and North America experience aviophobia, depending on the definition used [1].

Specific phobias are believed to be acquired through classical conditioning [1]. Cognitive distortion theory is accepted as the etiology of anxiety disorders, which in turn informs the use of cognitive behavioral intervention as a treatment approach [3]. The neurobiological etiology of specific phobias suggests that fear processing in the amygdala is impaired, which leads to increased activity in response to threat stimuli [4-6]. This overactivation of the amygdala is associated with the development of anxiety disorders and is inhibited by gamma-aminobutyric acid (GABA) networks. GABAergic activity is thus important to consider in the treatment of aviophobia [7].

The current treatment of aviophobia involves the use of virtual reality exposure (VRE) therapy, which may include diaphragmatic breathing exercises [8-10]. One study suggests that the addition of visual feedback from physiological signals allows for the maintenance of long-term treatment gains [11]. To our knowledge, limited literature exists on the pharmacological treatment of aviophobia. Two case studies reported inadvertent treatment of aviophobia with fluoxetine, a selective serotonin reuptake inhibitor, used to treat major depressive disorder in both patients [12]. Selective serotonin reuptake inhibitors (SSRI), such as sertraline, were shown to decrease regional cerebral blood flow in the amygdala [4,13]. Benzodiazepines, such as clonazepam, act on GABA-A receptors present in the cortex and the limbic system to help reduce anxiety and fear [4,14]. Propranolol, a beta-blocker, reduces basolateral amygdala activity and may be beneficial in the treatment of specific phobias [4]. The present report outlines the successful treatment of a patient with aviophobia through the use of different pharmacological agents targeting different receptors and components of aviophobia in conjunction with real-life gradual exposure therapy.

## Case presentation

The patient is a 29-year-old married, employed male of South Asian descent. The patient had no pertinent medical history or previous psychiatric history. Blood tests and routine labs assessed by the patient’s primary care physician were all within normal limits. On initial assessment, the patient presented with a chief complaint of "panic attacks.'' The first panic attack happened five years ago on a plane, where he experienced an episode of intense anxiety, palpitations, shortness of breath, and a feeling like "everything was closing in on him and that he was going to die." After that, he started avoiding flights, which significantly affected his personal, social, and work life. He started attending cognitive-behavioral therapy sessions about a year after this incident. Despite attending therapy regularly, he continued to experience severe anxiety and lived in constant fear of having a panic attack when traveling by air. He had been reluctant to consider medications due to concerns about side effects and the fear of becoming dependent on them. During the initial interview, the patient expressed significant frustration, particularly with his inability to travel by air. On mental status examination, the patient was awake, alert, and oriented to time, place, and person. He was well-groomed, maintained good eye contact, was cooperative and calm, and had no abnormalities in his movement, gait, or speech. His mood was anxious, with a congruent affect. The patient denied any hallucinations or delusions. His insight, memory, judgment, and concentration were all normal. He responded well to education about his condition and treatment with medications. He agreed with starting an initial low dose of sertraline. Over the course of three months, the dose of sertraline was titrated to 100 mg daily. During this time, he continued to attend sessions with his therapist.

With gradual improvement in general anxiety symptoms with sertraline, the patient requested a management plan to specifically target his fear of air travel. The patient agreed to consider gradual exposure therapy along with medications to help reduce anticipatory anxiety. The first step was to visit a small local airport to reduce his anxiety around planes. After gaining more comfort around planes, he was encouraged to try a discovery flight, which is an introductory flight where an individual flies in a small aircraft with a certified flight instructor. After completing this task, the final steps included a short commercial flight and then a flight of longer duration. To mitigate anxiety and fear associated with potential panic attacks during gradual exposure, he received a prescription for a combination of 10 mg propranolol and 0.5 mg clonazepam. These medications were advised to be taken before leaving his home, at check-in, and, if necessary, during air travel.

The patient had an initial goal of completing a three- to four-hour flight to another state. Despite experiencing some anxiety, he successfully completed a discovery flight without having a panic attack, effectively managing it with the use of medications. He felt incredibly inspired, and this motivated him to subsequently travel on a 45-minute commercial flight. This achievement marked a significant milestone, considering his inability to travel by air for over five years. He managed the flight without any issues using a combination of sertraline, propranolol, and clonazepam. After the first commercial flight, he developed further confidence in overcoming his fear. A few weeks later, he achieved his goal of flying to another state without feeling any anxiety. He felt assured that he had now fully overcome his fear of flying and should be able to manage long flights in the future. Although the patient was advised to continue to take propranolol and clonazepam before air travel, it was recommended that he try to use them less frequently over time as he develops more confidence. It is worth noting that his wife supported and accompanied him on all of the flights mentioned above. An important next step for the patient might be to attempt to fly alone to see how he would cope.

## Discussion

Aviophobia is a prevalent situational-specific phobia that can significantly limit aspects of a patient’s life. From feelings of embarrassment to relationship and career limitations, the negative consequences of having this phobia can be debilitating. The etiology and maintenance of specific phobias can be explained both from a neurobiological and psychological perspective. The use of psychopharmacologic drugs can aid in the various stages of treatment during exposure therapy. Benzodiazepines, such as clonazepam, help reduce anticipatory anxiety and can enable a patient to manage fear and anxiety during the initial stages of exposure therapy [15]. Low levels of serotonin (5-HT) in the amygdala could be part of the pathophysiology of specific phobias [4]. One study confirmed this via positron emission tomography, which illustrated decreased 5-HT1A receptor binding in both the amygdala and insula. [16]. SSRIs, which increase 5-HT levels in the synaptic cleft, can decrease panic sensations that are similar to panic attack disorder and may have broader potential use in situational-specific phobias, especially in patients with high motivation [15,17]. Beta-adrenergic blockers, such as propranolol, aid in the treatment of specific phobias by decreasing the perception of fear by reducing the symptoms of sympathetic arousal when confronted with the phobic stimulus and can be useful in managing acute fear and anxiety during gradual exposure therapy [15,18]. The cognitive behavioral theory model allows for the use of exposure therapy as a method for treatment [3], so VRE is often used to treat aviophobia [9]. In neurobiology, many studies indicate that the involvement of the limbic system and its dysfunction, specifically the amygdala, plays a key role in explaining the etiology of specific phobias [4].

The use of these pharmacologic agents has been studied in animal models. To our knowledge, the use of sertraline, propranolol, and clonazepam together during different stages of exposure therapy has not been examined; however, there are a few studies examining their individual effectiveness. One study compared the differences between VRE plus yohimbine hydrochloride, VRE plus propranolol, and VRE plus placebo over a period of two weeks in patients with specific phobias and found no significant differences [19]. It is important to add that none of the patients treated in the above study were mentioned to have aviophobia and only virtual reality therapy was examined.

There are strong elements in this case study’s gradual exposure protocols that are aligned with exposure response prevention (ERP) therapy, another form of behavioral treatment. Numerous studies continue to show the widespread effectiveness of ERP to treat anxiety-eliciting maladaptive behavioral disorders (Website: McClean. Everything You Need to Know About Exposure and Response Prevention Therapy, September 24, 2021). A key component of ERP therapy is the use of an exposure hierarchy, and this case study demonstrates a similar principle within the patient’s gradual exposures. Thus, the pairing of pharmacologic treatment with ERP therapy suggests an innovative opportunity to study the effectiveness of medication-assisted ERP. The use of medication-assisted ERP has been studied in other mental disorders aside from anxiety disorders. One study examined the treatment outcomes of patients diagnosed with obsessive-compulsive disorder and showed that the combination of medication and ERP had no significant treatment outcomes compared to patients treated with ERP alone but had better outcomes compared to patients treated with pharmacology alone [20]. Pharmacologic treatment can be valuable in conjunction with psychological treatment, whether it be gradual exposure therapy, extinction therapy, or another form of behavioral therapy. Further investigation and research is required to elucidate the effectiveness of the combination of gradual exposure therapy, sertraline, propranolol, and clonazepam in various stages of exposure therapy, as well as to determine how these drugs individually affect the theory and maintenance of aviophobia.

## Conclusions

Our report highlights the successful treatment of aviophobia in a 29-year-old male patient by combining psychopharmacological and behavioral therapies. It emphasizes the importance of collaboration between psychiatry and behavioral therapy in delivering patient-centered treatment for aviophobia. This information may help individuals with this condition access valuable treatment options online. However, further research is needed to explore and refine this treatment approach.
